# Automated Credibility Assessment of Web-Based Health Information Considering Health on the Net Foundation Code of Conduct (HONcode): Model Development and Validation Study

**DOI:** 10.2196/52995

**Published:** 2023-12-22

**Authors:** Azadeh Bayani, Alexandre Ayotte, Jean Noel Nikiema

**Affiliations:** 1 Centre de recherche en santé publique Université de Montréal et Centre intégré universitaire de santé et de services sociaux du Centre-Sud-de-l’Île-de-Montréal Montréal, QC Canada; 2 Laboratoire Transformation Numérique en Santé Montreal, QC Canada; 3 Department of Management, Evaluation and Health Policy School of Public Health Université de Montréal Montéal, QC Canada

**Keywords:** HONcode, infodemic, natural language processing, web-based health information, machine learning

## Abstract

**Background:**

An increasing number of users are turning to web-based sources as an important source of health care guidance information. Thus, trustworthy sources of information should be automatically identifiable using objective criteria.

**Objective:**

The purpose of this study was to automate the assessment of the Health on the Net Foundation Code of Conduct (HONcode) criteria, enhancing our ability to pinpoint trustworthy health information sources.

**Methods:**

A data set of 538 web pages displaying health content was collected from 43 health-related websites. HONcode criteria have been considered as web page and website levels. For the website-level criteria (confidentiality, transparency, financial disclosure, and advertising policy), a bag of keywords has been identified to assess the criteria using a rule-based model. For the web page–level criteria (authority, complementarity, justifiability, and attribution) several machine learning (ML) approaches were used. In total, 200 web pages were manually annotated until achieving a balanced representation in terms of frequency. In total, 3 ML models—random forest, support vector machines (SVM), and Bidirectional Encoder Representations from Transformers (BERT)—were trained on the initial annotated data. A second step of training was implemented for the complementarity criterion using the BERT model for multiclass classification of the complementarity sentences obtained by annotation and data augmentation (positive, negative, and noncommittal sentences). Finally, the remaining web pages were classified using the selected model and 100 sentences were randomly selected for manual review.

**Results:**

For web page–level criteria, the random forest model showed a good performance for the attribution criterion while displaying subpar performance in the others. BERT and SVM had a stable performance across all the criteria. BERT had a better area under the curve (AUC) of 0.96, 0.98, and 1.00 for neutral sentences, justifiability, and attribution, respectively. SVM had the overall better performance for the classification of complementarity with the AUC equal to 0.98. Finally, SVM and BERT had an equal AUC of 0.98 for the authority criterion. For the website level criteria, the rule-based model was able to retrieve web pages with an accuracy of 0.97 for confidentiality, 0.82 for transparency, and 0.51 for both financial disclosure and advertising policy. The final evaluation of the sentences determined 0.88 of precision and the agreement level of reviewers was computed at 0.82.

**Conclusions:**

Our results showed the potential power of automating the HONcode criteria assessment using ML approaches. This approach could be used with different types of pretrained models to accelerate the text annotation, and classification and to improve the performance in low-resource cases. Further work needs to be conducted to determine how to assign different weights to the criteria, as well as to identify additional characteristics that should be considered for consolidating these criteria into a comprehensive reliability score.

## Introduction

Along with the tremendous progress in internet technology the tendency to seek web-based health care–related information is increasing [1-3]. The literature emphasizes the significance of web-based information as the primary and foremost source of patient information [2,4-6]. Furthermore, younger adults are more inclined to seek their health care knowledge on the web [5]. Indeed, access to web-based health information empowers patients to take an active role in managing their health, equipping them with the knowledge they need to make informed decisions about their well-being [7].

However, maintaining a discerning approach when using health-related information from the internet is essential, as there exist potential risks that can undermine the well-being of internet users seeking health care guidance. Indeed, individuals may encounter misleading or wrong information [8,9]. Thus, web-based medical information for the public must be reliable (trustworthiness of the source and content's accuracy and timeliness). The “Infodemic” of misinformation is a term coined by the World Health Organization (WHO) to refer to the overwhelming activity of misinformation (but also disinformation) that makes it difficult for the general population to find reliable information. As evidenced, by its impact on the spread of COVID-19, misinformation limits the capacities of public health responses to diseases [10]. Ensuring reliance on trustworthy information validation sources is increasingly important, especially in light of the growing use of generative artificial intelligence which can sometimes provide inaccurate information [11]. Therefore, it is crucial to be able to identify trustworthy sources in order to obtain or verify any information.

Recognizing the significance of trustworthy web-based sources, several scoring systems and tools have been established to evaluate website trustworthiness [12,13]. These scoring systems sometimes have common attributes (or criteria). Examples of such systems include the Health on the Net Foundation Code of Conduct (HONcode) [1], the benchmarks set by the JAMA (*Journal of the American Medical Association*), and the DISCERN instrument [12]. One of the most widespread methods is the HONcode certification, which a website displaying health content must adhere to in order to obtain certification. The HONcode was created as a practical solution to help internet users recognize reliable health-related information on the internet while distinguishing it from potentially erroneous or hazardous content. This is particularly crucial given the widespread availability of web-based health information. The HONcode has been translated into numerous languages, allowing websites in various linguistic regions to adhere to its standards [1]. Acquiring HONcode certification requires voluntary submission which may depend on the decision of website owners. The certification process ended in 2022, but the criteria have been used in the literature to manually assess the trustworthiness of websites, chatbots, and teleconsultation services [14-16].

However, trustworthiness checking is highly associated with human perception since it is a complex and multidimensional process. Therefore, in order to assess the trustworthiness of web-based information, first it is necessary to operationalize the definition of scoring criteria using specific metrics and heuristics [17]. In addition, manually assessing a website’s adherence to scorings criteria is a tedious, costly, and time-consuming task and requires substantial human effort.

Several studies have implemented approaches to automate the detection of HONcode criteria on websites [1,18]. However, studies aiming to automate the process have only employed traditional machine learning (ML) methods for text classification such as support vector machines (SVM), naïve bayes (NB), and random forests (RF) [1,19-21]. In comparing the NB and SVM models, each demonstrated unique strengths in classifying websites based on the HONcode checklist. While the SVM model showed superior performance in recall, indicating a higher capability to identify relevant cases, it was less accurate than the NB model in the implementation by Boyer and Dolamic [18]. However, in subsequent applications by Boyer et Dolamic [21] focusing on specific HONcode criteria such as authority, complementarity, date, confidentiality, and transparency, SVM outperformed NB. This suggests that SVM has a stronger ability to effectively handle these specific aspects of website classification. Further, RF model had good performance on feature sets with many categorical variables [21,22].

Therefore, we have selected SVM and RF model due to their popularity and their better performances comparing to other ML approaches [1,18,19,21] in the previous studies. However, to the best of our knowledge no previous study has used attention-based models to automate reliability assessment considering HONcode criteria.

Indeed, recently, thanks to the progress in computing power, several state-of-the-art approaches have been introduced that, compared to traditional ML methods, do not necessitate vast amounts of training data for adequate performance. Examples of such approaches include Transformers (eg, Bidirectional Encoder Representations from Transformers [BERT]) [23,24]. Transformers are deep neural networks that use an attention-based mechanism that act on various natural language processing (NLP) tasks, such as supervised text classification, outperforming traditional models [25]. Both ML approaches and transformers analyze annotated text corpora to extract textual features for automated classification, each having its own strengths and weaknesses. Specifically, ML-based text classification tends to excel in scenarios with extensive text volumes. Transformers, often operate on a statistical basis and 1 advantage of them over traditional ML methods is their ability to perform well without requiring massive amounts of data. However, using them may lead to a trade-off: while we may sacrifice some precision, we can enhance recall [23].

In this study, we aimed to automate the assessment of HONcode criteria using different NLP approaches. We compared the performance of traditional ML models of RF and SVM with the pretrained transformer model of BERT, which is one of the state-of-the-art approaches in NLP.

## Methods

### Data Sets

Roughly 1000 web pages were provided by *Factually Health* company on January 31, 2023, a company that identifies reliable health-content websites. The web pages were manually cleaned, the redundant web pages were removed, also there were web pages that could not be used for the research because most of their contents were in a video, were related to a clinical study, were more anecdote than health paper, or did not allow python libraries to extract the information, which were deleted from the list. Finally, a data set of 538 factual web pages (from 43 verified websites) was included in this study. In total, 7 different diseases were covered by the web pages: arthritis, chronic obstructive pulmonary disease, COVID-19, hypertension, lung cancer, prostate cancer, and diabetes. Based on the URLs of each web page, each content was extracted as text files using the “justext” library in Python (Python Software Foundation) which removes the additional links and contents from websites, such as navigation links, headers, and footers.

### HONcode Criteria

An operational definition needs to be provided for each criterion, and the scope of application for each criterion is clearly defined to allow a standard annotation process. In Table 1, we defined two levels of criteria: (1) web page–level criteria that are specific to individual web pages and then may vary from one page to another within the same website (authority, complementarity, attribution, and justifiability); and (2) website-level criteria that are criteria applicable to the entire website (confidentiality, transparency, financial disclosure, and advertising policy).

Providing an operational definition involves identifying content on the website that can support the validation of the criterion. For the attribution, 2 elements should be extracted (references of the source of data and date of modification). Furthermore, ensuring justifiability in practice entails offering suitable references, which should concurrently fulfill the criteria for both attribution and justifiability. In addition, a balanced description of the health content regarding justifiability requires alignment with the prevailing scientific understanding of the disease (eg “*Manage diabetes effectively through regular monitoring, a nutritious diet, and guided medical consultations*” [balanced description] vs “*Unlock the secret to beating diabetes with extreme diets and miracle herbal supplements*” [unbalanced description]) that needs to be done more efficiently in coordination with a *fact-checking activity* (content's accuracy) and for this reason is not to be included in this step of assessing trustworthiness. Therefore, we extracted the authority, complementarity, attribution (only the date of the last update), and justifiability (only the references at the bottom of the page) from web pages' contents. The way content can be considered as validating criteria is described in Table 1.

Table 1 represents the 8 principles of HONcode and their operational description used to perform the manual annotation. The elements of validation represent the content of the website that can be annotated as fulfilling the criteria requirement.

**Table 1:** HONcode^a^ criteria: description and operationalized definition (elements of validation) for annotation.

Criterion	Description	Elements of validation	Level
Authority	Medical content on the website should only be written by a qualified professional unless, in a clear part, they explain that the advice is not written by a medically qualified individual or organization	Author or content reviewer names and degrees (eg, medically reviewed by Joe Doe, MD)	Web page
Complementarity	The information is provided only to support, not replace, the relationship between a patient and a doctor	References to 911 or references to a doctor (eg, consult your health care provider before taking this supplement)	Web page
Confidentiality	A sentence that mentions the usage of cookies or personal data relating to individual patients and visitors to a website	Web page containing a section describing the use of cookies and personal data (eg, the about-privacy-policy pages in the websites)	Website
Attribution	Providing references or links to the source of data of the content. Further, the date of the last modification should be clearly presented (eg, at the bottom of the page)	The date of the last update of the web page	Web page
Justifiability	For each reference, indicate if the reference is from a book, a scientific paper, or another website. Justifiability also requires a balanced description of the health content	The scientific references for a sentence or at the bottom of the web page. For example, (Arthritis. 2020)	Web page
Transparency	Websites provide easy and straightforward navigation or access to the information and provide contact addresses for visitors to seek further information or support	Web page containing sections for contact addresses and support information (eg, contact us)	Website
Financial disclosure	Any support including the identities of commercial and noncommercial organizations that have contributed funding, services, or material for the website should be clearly explained	Web page containing a section for financial disclosure information (eg, about-our-sponsors pages in the websites)	Website
Advertising policy	Clearly explained if advertising is a source of funding and the advertising policy adopted by the website owner	Web page containing section advertising policy (eg, the about-advertising-policy pages in the websites)	Website

^a^HONcode: Health on the Net Code of Conduct.

### Implementation Steps

**Figure 1:**
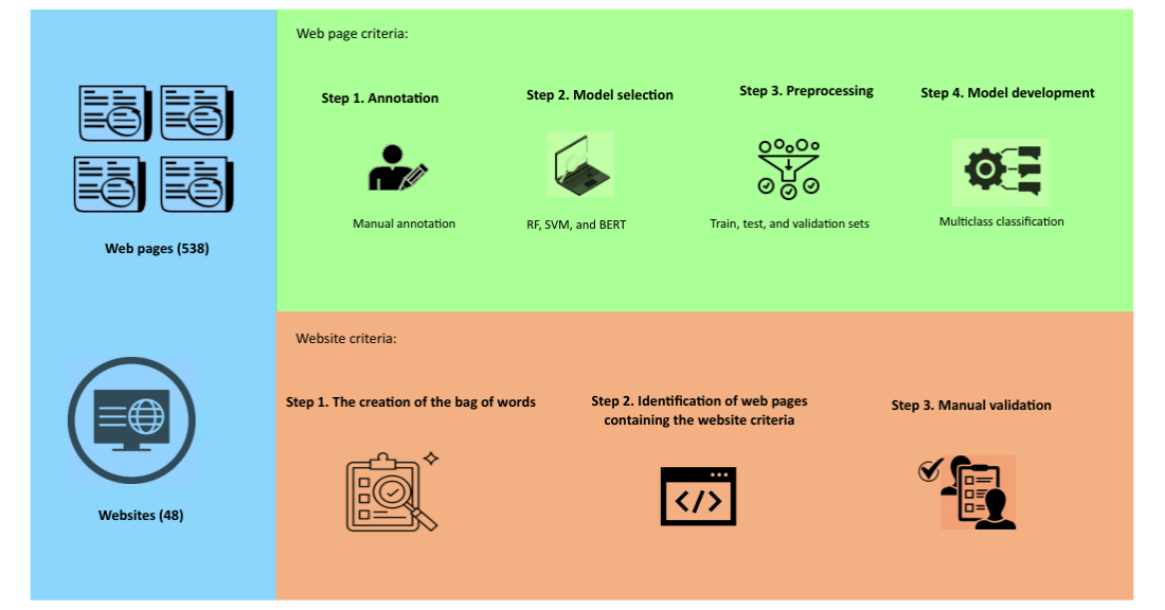
A summary of the implementation process. BERT: Bidirectional Encoder Representations from Transformers; RF: random forest; SVM: support vector machines.

In total, 4 steps were followed for the implementation process of web page–level criteria assessment, and 3 steps were used for website-level criteria assessment (Figure 1).

The 4 steps for the assessment of web page–level HONcode criteria are the following:

1. The annotation: we considered an initial seed of preannotated data to be used as the training data set to implement the classification models. According to Fernando and Tsokos [26], the manual annotation was continued until achieving a balanced representation of each criterion in terms of frequency, ensuring nearly equal counts for complementarity, authority, justifiability, and attribution using the portable serverless text annotation tool MedTator (version 1.3.11) [27]. Out of 538 web pages, 200 web pages contents were independently annotated by 2 of the authors (AB and AA) sentence-by-sentence, any disagreements between the authors were resolved by the third author. The remaining 338 web pages were set aside for automatic annotation. The total number of sentences in the 538 pages were 134,969, and the selected 200 web pages among them contained 47,886 sentences. The manual annotation for the web page level criteria—complementarity, authority, justifiability, and attribution generated 8579 sentences: 2996 sentences for*neutral*, 986 sentences for *complementarity,* 1763 for *authority*, 1274 for *justifiability*, and 1560 for *attribution*. Neutral sentences are sentences that do not correspond to any of the HONcode criteria.

2. Model selection: in total, three models have been used for the implementation of the learning process: (1) two classic algorithms RF and RF, and (2) the BERT model.

- The RF classifiers are suitable to effectively handle high-dimensional data and exhibit high performance even with imbalanced and noisy data [28]. The model combines multiple decision trees to make predictions.
- The SVM is a supervised model that, in many cases, exhibits effective and stable performance [29]. This model considers a representation of the training samples as points in space and maps each point to each class by a wider margin. Further, new samples are mapped into the class spaces that are predicted based on which side of the margins they belong.
- The BERT model: BERT is a bidirectional deep transformer encoder model pretrained on a huge general domain corpus from general English corpus from Wikipedia and Books Corpus [24]. A key benefit of BERT is its ease of use, achieved by simply incorporating a single output layer into the existing neural architecture in a fine-tuning process which means further training the model on a specific downstream task. The model has outperformed many existing models in various NLP challenges such as text classification [25].

3. Preprocessing: since the websites contained information about different diseases, to eliminate any bias in the data set, we tried to shuffle our data in order to include all the annotated diseases in the train, test, and validation set, and fed them to the models. The obtained performance was not affected by the type of the diseases, so we randomly shuffled the data set without taking into account the type of disease. The initial data format was plain text. Initially, we performed common text preprocessing tasks, which encompassed the removal of stop words, converting all sentences to lowercase, and applying a stemming technique to normalize the text data. Furthermore, to handle class imbalance, we implemented an undersampling technique. Specifically, we reduced the data for each criterion to match the size of the criterion with the fewest samples, which was set to “n=980” for uniform representation across all criteria. In addition to these preprocessing steps, we employed the TF-IDF (Term Frequency–Inverse Document Frequency) vectorization method using the TfidfVectorizer function in Python with the following hyper-parameters to convert sentences into numerical vectors:

TfidfVectorizer(max_df=0.75, max_features=1000, lowercase=True, ngram_range=(1,4))

For the BERT model, we opted for the “bert-base-uncased” tokenizer and a maximum sequence length of “128” to standardize the size of each input sentence. The model was initially trained with 12-layer, 768-hidden layers, with 110M parameters. To improve the performance, the hyper-parameters of the BERT were optimized using the Bayesian optimization approach in the “BayesianOptimization” library of python. This technique derives from Bayes’ theorem which is effective in computationally expensive problems. Bayesian Optimization aims to efficiently search for the optimal solution or parameter settings for a given problem while minimizing the number of costly evaluations. It does so by iteratively updating a probabilistic model to determine the most promising points in the search space, effectively reducing the number of expensive function evaluations required to find the best solution [30]. The hyperparameter tuning spaces are detailed in Table 2. Training involved a linear learning rate warm-up. The learning rate of 2 × 10^–6^, weight decay of 0.001, and a batch size of 16 were selected and with 2 epochs the model obtaining good performance.

**Table 2:** Hyper-parameter tuning search space.

Hyper-parameters	Range	Best trial
Learning rate	10^–7^, 10^–2^	2 × 10^–6^
Weight decay	10^–5^, 10^–1^	10^–3^
Number of epochs	1:5	2
Batch size	8, 16, 32, 64	16

An additional preprocessing step has been undertaken to enhance the complementarity criteria. Regarding this criterion, even if one could detect the presence of sentences that support the relationship between the patient and a doctor, it may not be enough. Indeed, a website that attempts to replace the relation between patient-doctor will be more harmful than the one that just forgets to respect the complementarity criterion. For example, sentences like “dermatologists’ opinions matter, but our content can provide you with alternative views,” should be considered negative. On the other hand, a positive example could be “the information on this website is for informational purposes only; consult a doctor if you experience any symptoms,” while a noncommittal example could be “your healthcare provider will use several tools to diagnose Rheumatoid Arthritis.” With this consideration, another pretrained BERT model was fine-tuned to analyze the sentiment of sentences that the previous model identified as related to complementarity. The list of annotated sentences has been leveraged to generate alternative sentences that convey complementarity with health care structures and professional sentiment through contextual word-embedding augmentation [31]. Additionally, text-generating augmentation has been applied using GPT-4 (Generative Pre-trained Transformer 4) to generate sentences expressing negative complementarity sentiment with health care structures and professionals. In other words, we ask GPT-4 to generate 250 sentences that do not respect the complementarity criterion of the HONcode certification. We then manually annotated each of them and removed those that were too redundant and repeated this process iteratively until we reached a point of saturation. These generated sentences have only been used to train and evaluate the complementarity model. Finally, 3 classes of negative, noncommittal, and positive with 156, 292, and 196 sentences were generated, respectively.

4. Model implementation, evaluation, and comparison: based on the manual annotation, we first implemented a multiclass classification for the criteria using the 3 models (SVM, RF, and BERT). The following hyper-parameters were used for the models:

We used the RandomForestClassifier method with the following hyperparameters: RandomForestClassifier(n_estimators=100, max_depth=5, random_state=0).

Further, the following method for SVM: LinearSVC(random_state=0, tol=1e-5) was used.

BERT: EPOCHS = 2, LEARNING_RATE = 2e-6, optimizer = AdamW(model.parameters())

A second step of training is performed for complementarity criterion using only the BERT model for multiclass classification of the complementarity sentences obtained by annotation and data augmentation (positive, negative, and noncommittal). To evaluate the performance of the models, the commonly used metrics in classification tasks—precision, recall, and *F*_1_-score—were used. Further, the confusion matrix and receiver operating characteristic curves of each model were plotted to access for the performance of each model. The remaining 338 web pages were annotated using ML models. We randomly selected 100 sentences by criteria for a manual review. For the manual review we computed the Cohen κ for 3 reviewers through a step-by-step process. Initially, we constructed a confusion matrix, representing the agreement and disagreement among the 3 reviewers. Next, we computed the observed agreement (Po), which signifies the actual agreement between the reviewers. Additionally, we determined the expected agreement (Pe), representing the agreement expected by chance. To derive Pe, we calculated the probability of each reviewer assigning a specific class by summing the probabilities across all classes and reviewers, followed by aggregating these probabilities for all classes.

In the final stage, Cohen κ was computed using the formula:

κ = (Po - Pe) / (1 - Pe)

The three steps for the assessment of website-level HONcode criteria are the following:

1. The creation of bags of words: a list of words and expressions for each criterion was collaboratively formulated, with an added set of words and expressions generated using GPT-4. The final word set for each criterion was validated by consensus (AA and JNN; Multimedia Appendix 1).
2. Identification of web pages containing the website criteria:
  using the bag of words for each criterion, we developed a custom algorithm to search for these words across all pages of each website. First, we conducted a search on the website's home page and extracted all the links leading to different pages of the same website. We initiated the process by looking for keywords that matched the criteria within the web page title. If unsuccessful, the search was expanded to encompass the content of the web page. In cases where this initial step did not yield any matches, we extended the search to encompass all the web pages of the respective website. Throughout this procedure, we prioritized the page that housed the greatest number of keywords pertinent to the specific criteria (Multimedia Appendix 1).
3. The manual validation: the validation of the web page containing the criterion is manually realized by consensus (AA and JNN). We considered the algorithm nonaccurate when it failed to retrieve a web page, or when the reviewers found the retrieved web page irrelevant to the criteria.

### Ethical Considerations

This research project, which exclusively uses publicly available data and does not involve human or animal subjects, is exempt from requiring ethical approval. It meticulously complies with established data privacy standards, ensuring no breach of confidentiality or privacy. Furthermore, the project does not entail any direct interventions or interactions with individuals, thus minimizing potential ethical concerns. The research committee of the University of Montreal has thoroughly reviewed our methodology and confirmed that this study falls outside the purview of the Medical Research Involving Human Subjects Act, further reinforcing its adherence to ethical guidelines.

## Results

### Website Criteria Identification

A list of 21 expressions (or words) has been identified for confidentiality, 21 for transparency, 30 for financial disclosure, and 29 for advertising policy (Multimedia Appendix 1). The algorithm used to identify website criteria was able to retrieve web pages with an accuracy of 0.97 for confidentiality, 0.82 for transparency, 0.51 for financial disclosure, and 0.51 for advertising policy. While on most websites the process leads to the selection of a single web page, some websites provide multiple pages that correspond to the same criteria (eg, different pages describing financial donations). In addition, many websites from well-known organizations do not disclose sponsorship information or provide revenue disclosures on their sites.

### Web Page Criteria Identification

#### Identification of the Criteria in the Web Page

Table 3 summarizes the prediction performance of the different models for each criterion in the web page level including precision, recall, and *F*_1_-score.

According to Table 3, for classifying the *complementarity* linear SVM had the highest precision and *F*_1_-score. For the neutral sentences and the other 3 HONcode criteria—authority, justifiability, and attribution—BERT exhibited higher *F*_1_-scores.

RF exhibits a discrepancy in its performance across the different classes. It demonstrates proficiency in 1 class—attribution—while displaying subpar performance in the other classes. BERT and SVM have a stable performance across the different classes. BERT has a better area under the curve (AUC) of 1.00 for authority, justifiability, and attribution. The global better performance for the classification of complementarity is validated by the confusion matrixes and the AUC that is equal to 0.99. As shown by the confusion matrixes tables, the complementarity sentences not identified by the BERT model have been classified as neutral sentences.

**Figure 2:**
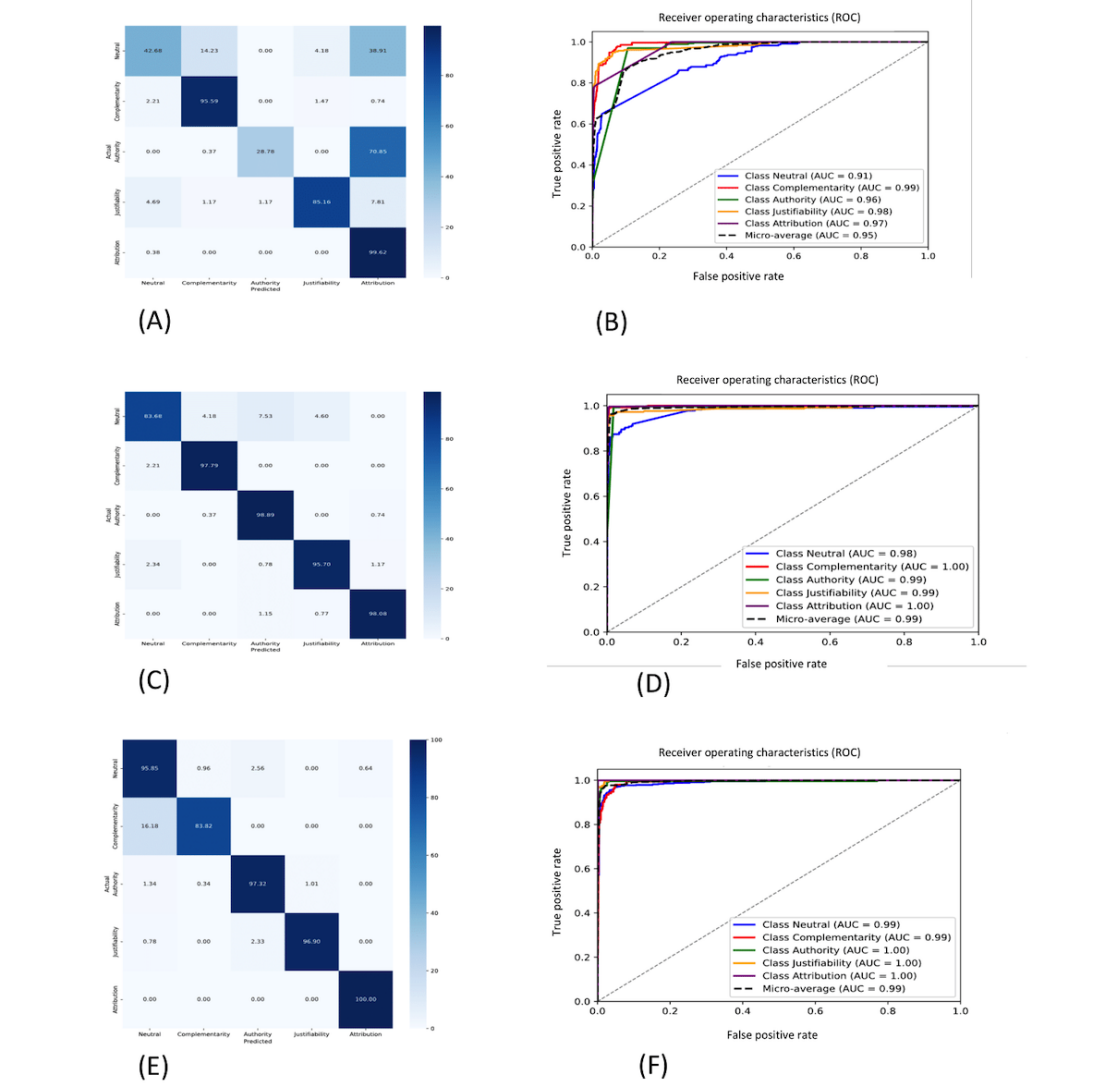
Confusion matrixes and the ROC curves for (A,B) RF model, (C,D) SVM model, and (E,F) BERT model. (A) Confusion matrix for RF model, (B) ROC curve for RF model, (C) confusion matrix for SVM model, (D) ROC curve for SVM model, (E) confusion matrix for BERT model, and (F) ROC curve for BERT model. AUC: area under the curve; BERT: Bidirectional Encoder Representations from Transformers; RF: random forest; ROC: receiver operating characteristic; SVM: support vector machines. For a higher-resolution version of this figure, see Multimedia Appendix 2.

Figure 2 illustrates the confusion matrixes and the receiver operating characteristic curves for the 3 models.

**Table 3:** Models prediction performance for web page–level HONcode^a^ criteria.

Targets or model	RF^b^				SVM^c^				BERT^d^		
	Precision	Recall	*F*_1_-score	Precision		Recall	*F*_1_-score	Precision		Recall	*F*_1_-score
Neutral	0.84	0.43	0.57	0.94		0.84	0.89	0.92		0.96	0.94
Complementarity	0.87	0.96	0.91	0.96		0.98	0.97	0.97		0.84	0.90
Authority	0.96	0.29	0.44	0.92		0.99	0.95	0.96		0.97	0.97
Justifiability	0.94	0.85	0.89	0.95		0.96	0.95	0.98		0.97	0.97
Attribution	0.46	1.00	0.63	0.98		0.98	0.98	0.99		1.00	0.99

^a^HONcode: Health on the Net Code of Conduct.

^b^RF: random forest.

^c^SVM: support vector machine.

^d^BERT: Bidirectional Encoder Representations from Transformers.

#### Complementarity Prediction

**Figure 3:**
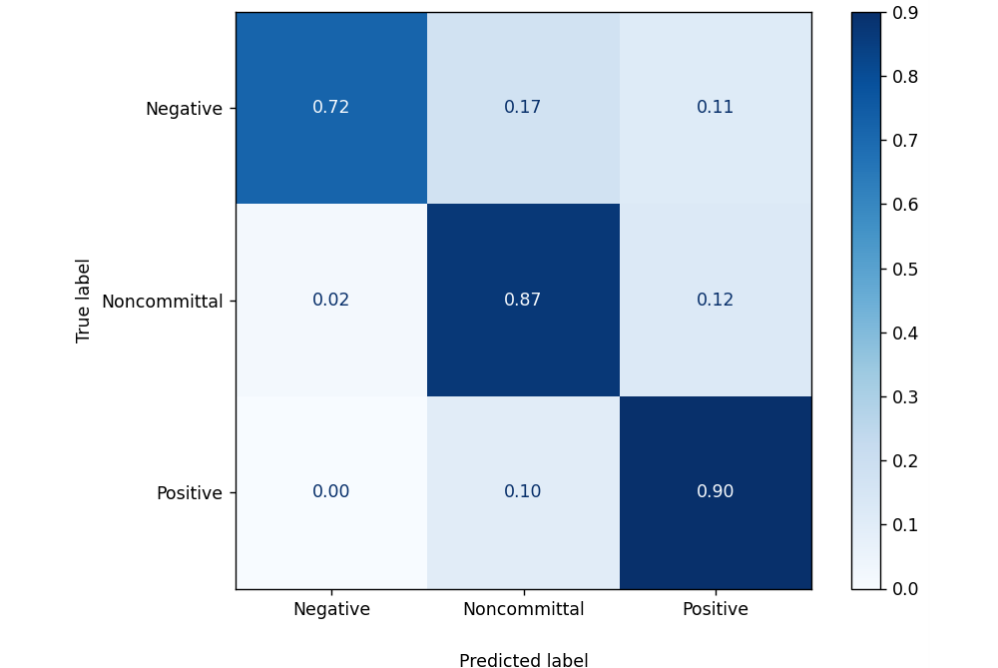
Confusion matrix of the complementarity model.

Figure 3 shows that our complementarity model got a recall of 0.72 for the prediction of the negative sentences and a recall of 0.87 and 0.90 for the prediction of the noncommittal and the positive sentences respectively. On the other hand, the precision is 0.97, 0.76, and 0.80 for the prediction of the negative, noncommittal, and positive sentences, respectively. Overall, the model got a balanced accuracy of 0.83.

#### Final Evaluation of Web Page Criteria

The ultimate model was deployed on web pages that were not included in the training phase. The outcomes were documented in an Excel file and subjected to manual validation by the 3 authors (JNN, AA, and AB). For each author, 100 common randomly selected sentences were mutually evaluated with their corresponding predicted labels. A few discrepancies were observed between the reviewers. Most of them were related to the 2 criteria of justifiability and complementarity. For example, 2 reviewers perceived the following sentence as a complementary suggestion while the third reviewer categorized it under a neutral criterion: “However, wheezing can also be a symptom of lung cancer, so it’s a good idea to bring it to your doctor’s attention.” The resulting precision for the sentences was determined to be 88%. Furthermore, a Cohen κ score of 81.78% was computed among the 3 reviewers.

## Discussion

### Principal Results

The objective of this study was to automate the evaluation of the HONcode criteria aiming to determine the credibility of health-related websites. In this research the data was collected from reputable sources of health care, by “Factually Health” Company. The data collection process involved selecting 8 different diseases which reflected a diverse range of health-related topics, ensuring that the data set encompasses various aspects of health information. Further, a rigorous data cleaning process was undertaken, including the removal of redundant links, web pages with video content, clinical studies, or anecdotal content which enhanced the scientific quality of the data by eliminating irrelevant or low-quality sources.

Our approach reflects various initiatives in the field, where the HONcode has been applied to a range of methods and web-based sources. Similar to these efforts, we have used traditional ML models in NLP tasks, specifically focusing on automating the detection of HONcode criteria. [1,18,19,32,33]. However, using the advantages of fine-tuning pretrained models in many NLP cases, such as text classification, showed a higher performance [22,24,25]. Due to the large amount of textual data in the websites, implementing any ML model requires text annotation which is an expensive and time-consuming task [1,18,34]. Therefore, the process often engages semisupervised and classification techniques [34]. In this study, we implemented the ML approaches to classify and annotate the health care–related contents in the websites. The yielded performances revealed intriguing insights into the classification abilities of the models.

As the models are intended for real-world application on unstructured data from health-related websites, their input will mainly comprise plain text extracted from website content. To accommodate this variability and improve the generalizability, a class labeled neutral has been introduced. This class allows the model to effectively classify the unlimited text content extracted from these websites. Previous studies [1,18,21,32,35,36] did not incorporate a neutral class in their analysis and predominantly relied on structured data formats which might pose challenges in the generalizability of their findings. In the neutral class, all models exhibit good precision values, ranging from 0.84 to 0.94, signifying their accuracy in classifying instances. However, a noticeable discrepancy emerges in recall scores, varying from 0.43 to 0.84. This discrepancy suggests that while these models excel in correctly identifying neutral instances, they tend to miss a substantial proportion of actual instances in this criterion. The reason could potentially stem from the diverse nature of sentences in this category since this class contained all the sentences that did not belong to the HONcode criteria.

The SVM model, demonstrated remarkable precision in certain criteria, such as authority (0.96), but a very low recall (0.29), indicating its proficiency in identifying authority instances while missing a significant portion of true positives. This aligns with findings by Boyer and Dolamic [18,21] who also reported underperformance in this criterion. The study [21] highlighted SVM's trade-off between recall (0.69) and precision (0.73), contrasting with NB, which exhibited higher recall (0.88) but lower precision (0.52). This discrepancy might be due to the criterion's complexity, demanding intricate feature extraction methods. The BERT model displayed superior performance across all metrics in this criterion, suggesting its capacity to classify the authority instances more effectively. Further, in line with previous studies SVM had better performance than other ML baselines [18,19,21,37]. In terms of the justifiability criterion, all models demonstrated strong performance, achieving precisions of 0.95 and 0.98, along with recalls of 0.96 and 0.97 for SVM and BERT, respectively. However, in contrast to our findings, a prior study [19] excluded this criterion due to its challenging nature in detection. Another study [21] reported a precision of 0.69 and a lower recall of 0.58, emphasizing the intricate classification demands posed by the justifiability criterion.

Notably, in the attribution category, all models achieve high precision scores. The higher recall scores, particularly for SVM and BERT, reaching a perfect 1.00, underscore their ability to capture nearly all actual instances of the attribution criterion. Several previous studies [18,32,36] reported higher precisions (0.83 to 0.94) among all the criteria for the Attribution which are consistent with our results for this criterion.

We also fine-tuned a multiclass BERT model for the complementarity criterion. All the previous studies considered complementarity as a binary class prediction of yes or no [1,21,32,36] and although their results were feasible with the precisions from 0.84 to 0.96, however given the complex nature of this criterion's interpretation, the BERT model yielded the precision of 0.97, 0.76, and 0.80 for the prediction of the negative, noncommittal, and positive sentences, respectively. This refined approach demonstrated performance levels that are comparable to those found in existing literature [38,39] however, more research for augmenting the training data set and improving the performance of models for the complementarity criteria is recommended. Regarding the AUCs, for the complementarity criterion the SVM model had a better performance. For the 4 other criteria of neutral, authority, justification, and attribution, the BERT model had the best performance. Furthermore, although the κ value is satisfactory but not exceptional, it underscores the inherent challenges associated with the implementation of the criteria and emphasizes the models' competent performance considering the complexity of automating the identification of criteria. The findings of this study, emphasize the strengths and weaknesses of each model across different criteria. While BERT consistently demonstrated robust and reliable performance, other models exhibit varying strengths, excelling in certain categories but showing limitations in recall for specific classes. These performance variations underscore the importance of model selection based on the specific task requirements, prioritizing precision, recall, or a balanced trade-off between the 2 metrics for each category [40].

The criteria for evaluating the overall trustworthiness of a website can be considered as reputation-related criteria. These are not directly linked to specific pages and should be evaluated independently from page-level criteria. Using a bag-of-words analysis facilitates an effective evaluation of these criteria [41]. Nevertheless, the information regarding sponsorship and advertising funding for the site is not always readily available. The performance of the algorithm does not necessarily indicate a shortcoming, but rather it might simply mean that there is no relevant information available to retrieve. Indeed, many websites of well-known organizations, including those run by public and international organizations, do not disclose this information openly on their platforms. While these 2 criteria might be pertinent for websites owned by private entities, applying them to public and international organizations could potentially undermine the site's overall score. Consequently, this might not accurately reflect the website's true level of trustworthiness. Further work needs to be conducted to determine how to assign different weights to the criteria, as well as to identify additional characteristics that should be considered for consolidating these criteria into a comprehensive reliability score.

### Limitations and Future Work

One of the primary limitations of this study is the use of a simple bag of words for detecting website-level criteria. The process can sometimes be time-consuming for certain sites when it requires browsing through thousands of pages for a single website in order to identify the page of interest. Furthermore, it is important to note that these keywords will need to be updated regularly to maintain their effectiveness. In further works, we will develop a substantial database through the use of this algorithm and initiate an automated learning process for website-level criteria.

In addition, our complementarity model generally showed significant performance in sentiment prediction, but it still has the imbalance in our data set. In fact, we could not generate more negative sentences with GPT-4 without redundancy, and it was also more difficult to find examples of this class in web-based sources. Moreover, several negative sentences were way more ambiguous and complex to classify than those in the positive and noncommittal classes of our data set. Therefore, finding other sources for the negative class of our data set may improve the performance of the sentiment model for complementarity classification.

Since the lack of a suitable data set for implementing automated classification models is often considered as a major limitation, exploiting the power of transformers to generate the synthetic data, is suggested for future research. By augmenting the initial data set, we can provide a reliable way to improve the training data set and the performance of models. However, further studies on other pretrained transformers to analyze their performance and optimize their performance for the task will be beneficial.

### Conclusions

The findings showed the potential of automating the assessment of HONcode criteria in web content using different ML approaches. Our results suggest the efficiency of fine-tuning pretrained models such as the BERT model in the task of text annotation for assessing the reliability criteria of websites, especially in cases where the initial annotated data set is limited [42]. Regarding the criteria of authority, justifiability, and attribution, the BERT model achieved higher performance compared to traditional approaches using RF and SVM. The overall approach could be used to automate HONcode criteria detection, accelerate the text annotation and classification, and to improve the performance of the models in low-resource cases.

## Appendix

Multimedia Appendix 1 Bag of keywords list.

Multimedia Appendix 2 Confusion matrixes and the ROC curves for (A,B) RF model, (C,D) SVM model, and (E,F) BERT model. (A) Confusion matrix for RF model, (B) ROC curve for RF model, (C) confusion matrix for SVM model, (D) ROC curve for SVM model, (E) confusion matrix for BERT model, and (F) ROC curve for BERT model. AUC: area under the curve; BERT: Bidirectional Encoder Representations from Transformers; RF: random forest; ROC: receiver operating characteristic; SVM: support vector machines.

## Abbreviations

AUC: area under the curve
BERT: Bidirectional Encoder Representations from Transformers
GPT-4: Generative Pre-trained Transformer 4
HONcode: Health on the Net Code of Conduct
JAMA: Journal of the American Medical Association
ML: machine learning
NB: naïve bayes
NLP: natural language processing
RF: random forest
SVM: support vector machine
TF-IDF: Term Frequency-Inverse Document Frequency
WHO: World Health Organization

## Data Availability

The data sets generated and analyzed during this study are available from the corresponding author on reasonable request.
