# Supplementary figures and images for: Automated Credibility Assessment of Web-Based Health Information Considering Health on the Net Foundation Code of Conduct (HONcode): Model Development and Validation Study
Source: JMIR Form Res. 2023 Dec 22;7:e52995. doi: 10.2196/52995 (PMC10770789; doi:10.2196/52995)

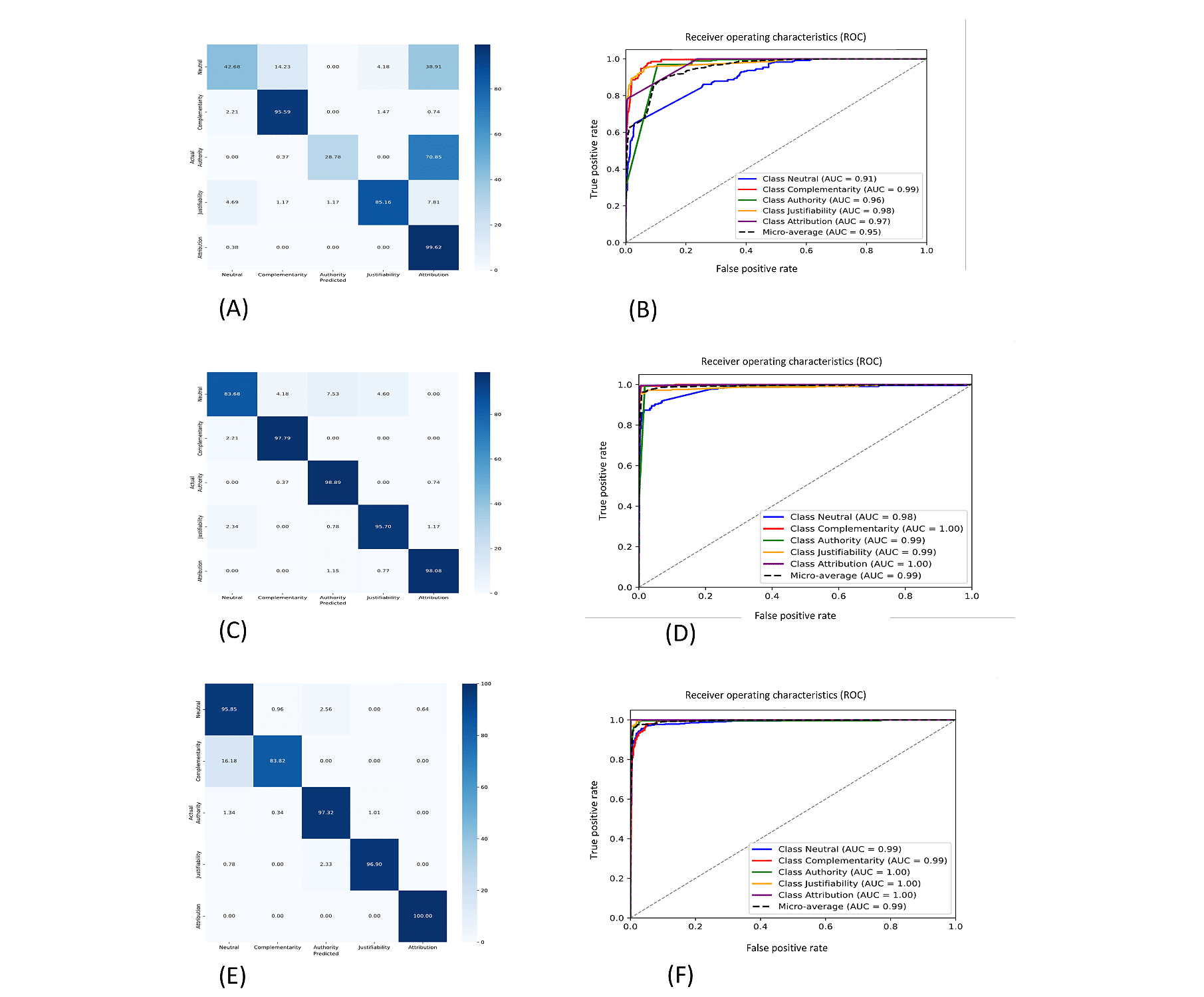

Supplement: Multimedia Appendix 2 [file formative_v7i1e52995_app2.png]
